# Pig-eRNAdb: a comprehensive enhancer and eRNA dataset of pigs

**DOI:** 10.1038/s41597-024-02960-7

**Published:** 2024-02-01

**Authors:** Yifei Wang, Weiwei Jin, Xiangchun Pan, Weili Liao, Qingpeng Shen, Jiali Cai, Wentao Gong, Yuhan Tian, Dantong Xu, Yipeng Li, Jiaqi Li, Jing Gong, Zhe Zhang, Xiaolong Yuan

**Affiliations:** 1 Guangdong Provincial Key Laboratory of Agro-Animal Genomics and Molecular Breeding, Guangdong Laboratory of Lingnan Modern Agriculture, National Engineering Research Center for Breeding Swine Industry, State Key Laboratory of Swine and Poultry Breeding Industry, College of Animal Science, South China Agricultural University, Guangzhou, 510642 China; 2 Hubei Key Laboratory of Agricultural Bioinformatics, College of Informatics, Huazhong Agricultural University, Wuhan, 430070 China

**Keywords:** Computational biology and bioinformatics, Genetics

## Abstract

Enhancers and the enhancer RNAs (eRNAs) have been strongly implicated in regulations of transcriptions. Based the multi-omics data (ATAC-seq, ChIP-seq and RNA-seq) from public databases, Pig-eRNAdb is a dataset that comprehensively integrates enhancers and eRNAs for pigs using the machine learning strategy, which incorporates 82,399 enhancers and 37,803 eRNAs from 607 samples across 15 tissues of pigs. This user-friendly dataset covers a comprehensive depth of enhancers and eRNAs annotation for pigs. The coordinates of enhancers and the expression patterns of eRNAs are downloadable. Besides, thousands of regulators on eRNAs, the target genes of eRNAs, the tissue-specific eRNAs, and the housekeeping eRNAs are also accessible as well as the sequence similarity of eRNAs with humans. Moreover, the tissue-specific eRNA-trait associations encompass 652 traits are also provided. It will crucially facilitate investigations on enhancers and eRNAs with Pig-eRNAdb as a reference dataset in pigs.

## Background & Summary

During the growth and development of organisms, the transcriptions of genes are subject to a variety of complex regulators^1^. Previous studies have reported that transcriptions are controlled by the *cis*-regulatory elements^2,3^. Enhancers, first discovered in 1981, are widely considered as the important *cis-*regulatory elements to significantly increase the transcriptions of target genes^4^, and have been shown to bind the promoters of target genes at a distance to activate transcriptions^5,6^, regardless of directions. It is important to note that enhancers are strongly specifically expressed in different cell types and tissues^7,8^. Recently, ENCODE^7^, FANTOM5^9,10^, and Roadmap Epigenomics^11^ projects have established to collect enhancers of humans and animals.

Recently, enhancers in a genome-wide manner have been identified with multi-omics in humans and mice by machine learning, which greatly decreases the time and cost to define enhancers, compared with experimental methods^12^. Currently, there are numerous strategies utilizing machine learning for enhancer prediction. For instance, in humans and mice, PEDLA uses a lot of heterogeneous data to predict enhancers in H1 cells, e.g., the chromatin accessibility (DNase-Seq), RNA-Seq, DNA methylation, the ChIP-Seq of 27 histone modification marks and 15 transcription factors (TFs), and achieved 97.7% accuracy by a deep neural networks algorithmic framework in 2016^13^. In 2017, He *et al*. develop REPTILE^14^ based on random forest classifier with the epigenomic signatures to predict enhancers, e.g., H3K4me1, H3K4me2, H3K4me3, H3K27ac, H3K27me3, and H3K9ac as well as DNA methylation, and the accuracy of REPTILE is 94.4%, which is higher than DELTA^15^ in H1 cells. In 2019, Ramisch *et al*.^12^ use two binary random forest classifiers for the enhancer prediction using the signatures of H3K4me1, H3K4me3, and H3K27ac, and yield stable results with the area under the precision-recall ∈ [0.91,0.95], which is superior to REPTILE^14^. In 2021, Zhanlin Chen *et al*. initiate DECODE utilizing STARR-seq data, chromatin accessibility (ATAC-seq and DNase-seq) and signatures for H3K27ac, H3K4me3, H3K4me1 and H3K9ac to extract accurate cell-type-specific enhancers^16^, and the average of the precise recall of area under the curve of DECODE is 24% higher than Matched-Filter^17^. These appearances suggested that multi-omics with machine learning are likely to improve the accuracy of enhancer prediction.

Enhancer RNAs (eRNA) are discovered and reported as the non-coding RNAs for bidirectional transcription dependent on RNA polymerase II in the enhancer region^18^. Previous studies have showed that 40,000–65,000 eRNAs expressed in humans, and eRNAs have been reported to regulate the transcriptions of target genes^19^ and the activation of enhancers^10,20^. For instance, eRNAs promote transcriptional condensation formation to activate enhancers in MCF7 breast cancer cells^21^, and the eRNA transcribed from *Pcdh*-*α HS5-1* enhancer is likely to form an R-loop structure and alter the chromatin structure to strengthen the expression of *Pcdh-α*^22^. Furthermore, several eRNA databases have been completed in humans^23,24^ and animals^25^. For example, HeRA^23^ characterizes the human eRNAs, which collects the RNA-seq files (9577 samples across 54 human tissues) from GTEx and the annotations of enhancers from ENCODE, FANTOM and Roadmap Epigenomics projects. GPIeR characterizes the impact of genetic variants on eRNA expression using large-scale omics data from The cancer Genome Atlas^26^. Besides, Animal-eRNAdb^25^ has been developed for the eRNAs of animals, e.g. chickens, sheep, rats and mice, basing on 5085 RNA-seq data from NCBI and the annotations of enhancers from SEA 3.0 and EnhancerAtlas v2.0.

The pig is not only an important agricultural animal that provides pork and animal proteins, but also serves as a necessary biomedical model for humans^27^. However, the eRNA profiles have not been characterized in pigs. In this study, we proposed a package CNNEE (a convolutional neural network (CNN)-based pipeline to track enhancers and eRNAs, https://github.com/WangYF33/CNNEE) using multi-omics of the chromatin accessibility (ATAC-seq) and histone modifications (H3K27ac and H3K4me3) in multi-tissues, as well as using the data of RNA-seq due that enhancers are able to transcribe eRNAs. Moreover, we collected RNA-seq data from 607 samples across 15 pig tissues to characterize the eRNA profiles, the target genes and regulators for eRNAs, the tissue-specific eRNAs, the housekeeping eRNAs (HKeRNAs), and the associations between eRNAs and phenotypes in pigs. Pig-eRNAdb will facilitate the functional investigations of enhancers and eRNAs in pigs.

## Methods

**Fig. 1:**
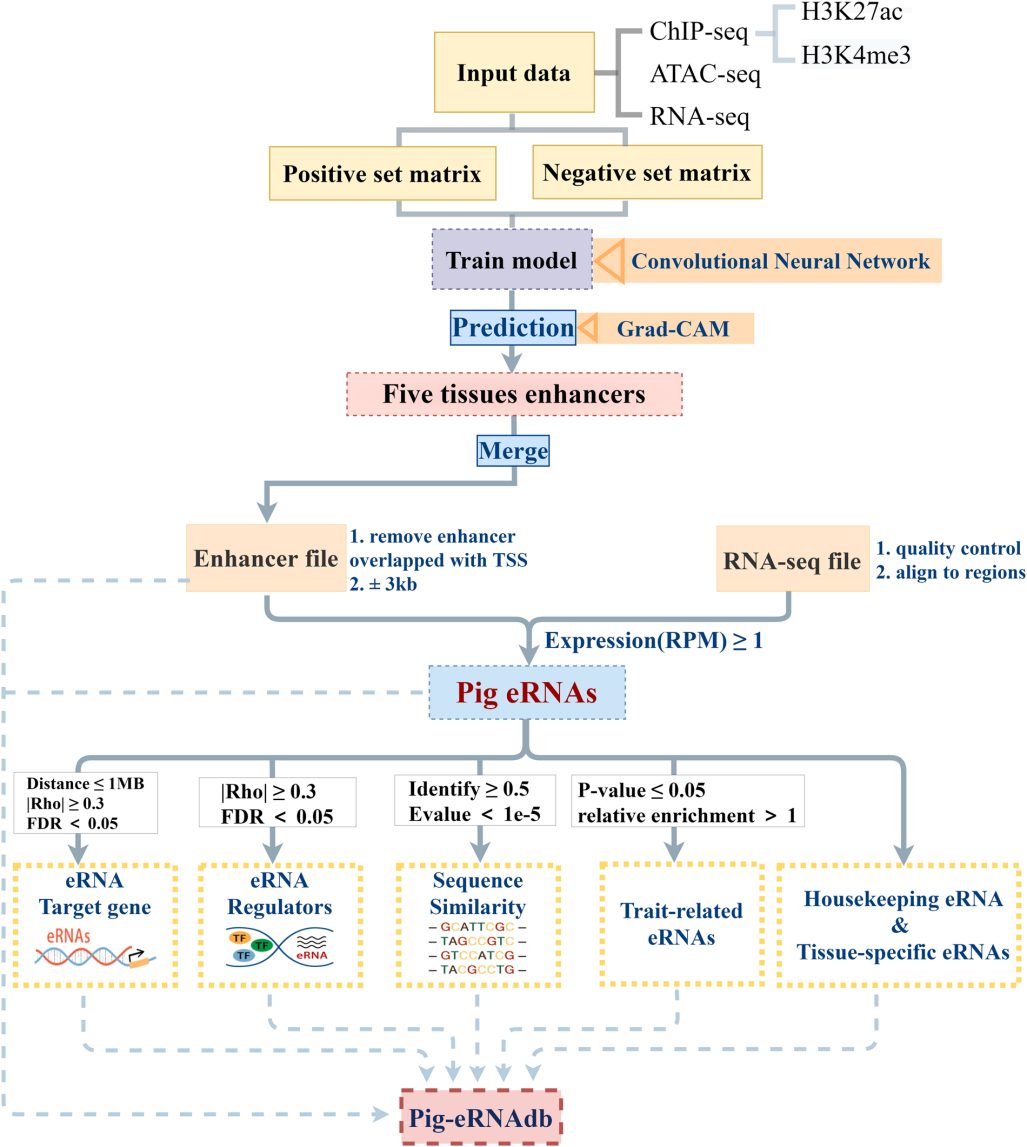
Overview of Pig-eRNAdb.

Pig-eRNAdb is an integrated dataset of enhancers and eRNAs, containing the coordinates of enhancers and eRNAs, the target genes and regulators of eRNAs, and the sequence similarities of eRNAs. The analysis protocols of Pig-eRNAdb were showed in Fig. 1.

### Data collection and processing

We tried to collect data from multiple developmental stages and tissues to achieve the comprehensive prediction of enhancers. In this study, we downloaded the bigwig and narrow peak files of ATAC-seq, ChIP-seq of H3K27ac and H3K4me3, as well as RNA-seq data across five tissues (heart, livers, spleen, muscle and fat) of pigs (Supplemental Table S1). Signal tracks in the bigwig files were extracted for ATAC-seq, ChIP-seq of H3K27ac and H3K4me3 by python (version: 3.6.4). 607 RNA-seq data were downloaded from the Sequence Read Archive (https://www.ncbi.nlm.nih.gov/sra) across 15 tissues of pigs (Supplemental Table S2), e.g., hearts, livers, spleens, backfat, gallbladders, jejunums, kidneys, lungs, ovaries, pituitaries, muscles, skeletal muscles (Sk-Muscle), longissimus dorsi muscles (Ld-Muscle), pineal and ileum. The raw data were downloaded and converted as fastq files using fastq-dump in SRA Toolkit (version: v2.8.2), and enslaved to quality control and clean with fastp (version: 0.23.0)^28^, and were mapped to the pig reference genome (https://ftp.ensembl.org/pub/release-105/fasta/sus_scrofa/dna/) using HISAT2 (version: v2.1.0)^29^ to obtain the bam files, which were sorted by SAMtools (version: 1.11)^30^. Furthermore, we merged all files using the merge parameter of STRINGTIE (version: 2.1.1) to unify the standard. Finally, we used STRINGTIE (version: 2.1.1) to compute transcripts per million (TPM)^31^ to normalize the gene expression, and obtained a gene expression matrix. The genome annotation file was download from Ensembl (https://ftp.ensembl.org/pub/release-105/gtf/sus_scrofa/). The annotations of enhancers in humans and mice were downloaded from ENCODE (http://screen.encodeproject.org/) (Supplemental Table S3). The repeat elements of pigs were downloaded from the UCSC (http://hgdownload.soe.ucsc.edu/goldenPath/susScr11/database/).

### Input data matrix construction

We used the multi-omics features of the RNA-seq data, the chromatin accessibility and the histone modifications (H3K27ac and H3K4me3) as input data. Firstly, the raw sequencing data from RNA-seq were processed into bam files according to the transcriptome data processing flow in the above method, and then used the bamCoverage tool from deepTools, and utilized the bigWigToBedGraph and bedGraphToBigWig tools from UCSC to process the bam files into bigwig files.

The peaks of H3K27ac and H3K4me3 intersected with the ATAC-seq peak region, and then intersected with transcripts with expression (TPM) greater than 0.1 as the narrow peak of transcript data. The ATAC-seq peaks overlapping with a transcript peak and the peaks of an active histone were defined as the active enhancers and positive training set. The negative training samples were selected from the background by downsampling, with 10 times of the positive set, and we unified the positive and negative set samples into a 4 kb window and aggregate the signals in 10 bp bins.

The input data was placed into a matrix of size 4 × 400 which contained signal trajectories for three epigenetic data and transcriptome data in the 4 kb region. In the input matrix, each value represented the signal strength at the corresponding genomic location. The precision of detected boundaries of enhancer regions was determined by the resolution of the signal integration. In this study, each value in the matrix of input data represented the average epigenetic signal value in a bin with a resolution of 10 bp.

### Construction of the predict model in pigs

We constructed a CNN-based binary classifier with reference to ResNet^32^ and DECODE^16^, including convolutional layers, pooling layers, and dense fully connected layers. The convolutional layer was responsible for comprehensively extracting features from multi-omics. The Seekeze-and-Excitation blocks for calculating residual features and adaptively recalibrating channel-based feature responses were placed among each convolutional layer^33^. The max-pooling layer reduced the number of parameters trained in the previous convolutional layer and preserved some important feature information. The pooled layers were then fed into the fully connected layer makes a sigmoid prediction about the probability of an enhancer in the region.

In the study, we discovered six models. Model 1: 6 convolutional layer, 1 max-pooling layer, 1 fully connected layer; Model 2: 7 convolutional layer, 2 max-pooling layer, 1 fully connected layer; Model 3: 6 convolutional layer, 2 max-pooling layer, 1 fully connected layer; Model 4: 7 convolutional layer, 3 max-pooling layer, 1 fully connected layer; Model 5: 1 convolutional layer, 1 max-pooling layer, 1 fully connected layer; Model 6: 5 convolutional layer, 1 max-pooling layer, 1 fully connected layer.

The positive set classification threshold criteria (threshold > 0.5) established by previous studies^12,14^ was utilized in the present study. To better understand the decision process of CNN and narrow the boundaries of candidate enhancer regions, we added the Gradient-weighted Class Activation Mapping (Grad-CAM) method^34^. Grad-CAM used gradient information flowing for last convolutional layer in the CNN to understand the importance of each neuron for category recognition and obtained a high-resolution subset of images of the most informative content with the target. Therefore, we used the global-average-pooled gradient of the positive set to generate importance scores for each activation map generated by the final convolutional layer, which were the weights of the linear combination of all corresponding activation feature maps. We multiplied the activation maps of the final convolutional layer by their respective weights and summed all the activation maps. We used ReLU function to retain the values which had a positive effect on the classification, and suppressed the values that showed a negative effect on the classification. Finally, depending on the importance score, we filtered out the regions on the positive set which were lower than the average of the importance scores.

### Enhancers and eRNAs annotation

We used BEDTools (version: 2.26.0)^35^ to remove the predicted enhancers which overlapped with the transcription start sites (TSSs) as the pig enhancers, and extended ± 3 kb from the midpoint of the enhancers, and defined these 6 kb regions as the potential eRNA regions, according to HeRA^23^ and Animal-eRNAdb^25^. To reduce the effect of the known protein-coding genes, we excluded the eRNA regions which were overlapped with the exons of protein-coding genes. Next, BEDTools (version: 2.26.0)^35^ was used to calculate the read counts per sample mapped in the eRNA regions, and normalized the read counts using the trimmed mean of M values from the edgR package of R (version: 4.1.0). Furthermore, the expressions of eRNAs were normalized by reads per million (RPM) method^36^. We defined the eRNAs with average RPM ≥ 1 in at least one tissue as the detectable eRNAs. The eRNAs of one certain tissue expressed more than 3 times of the average expression in other tissues were defined as tissue-specific eRNAs^37,38^.

### Comparison of sequence similarity and conservation between Pig and human eRNA

Previous studies have confirmed that enhancers are conserved between pigs and humans^39^. Therefore, we compared the sequence similarity and the degree of conservatism between pig and human eRNAs. Firstly, we downloaded the sequence file of the human eRNAs from HeRA^23^, and then used BEDTools (version: 2.26.0) to obtain the sequence file of pig eRNAs from the sequence file of the pig reference genome. Next, the similarity of each pig eRNA was calculated with all human eRNAs using blastn and specified that the similarity ≥ 0.5 and expectation value (*E*-value) ≤ 1e-5 was statistically significant. We used LiftOver (minMatch = 0.5)^40^ to screen for the pig eRNAs which were sequence conserved with human eRNAs. After the converted genomic version, the eRNA regions of pigs overlapped with the human eRNA regions were defined as functionally conserved with human eRNAs.

### Identification of target genes and putative regulators of pig eRNAs

In each tissue, the genes that closed to eRNAs (distance < 1 Mb) and significantly co-expressed with eRNAs were defined as potential target genes for eRNAs using Spearman’s correlation^23^. |Rho| ≥ 0.3 and *P*-value < 0.05 were defined as statistically significant. We performed the Gene Ontology (GO) and the Kyoto Encyclopaedia of Genes (KEGG) enrichment analysis on target genes of eRNAs using clusterProfiler package of R (version: 4.1.0). We collected annotations of pig TFs from AnimalTFDB 3.0 (http://bioinfo.life.hust.edu.cn/AnimalTFDB/)^41^, and extracted the expressions of TFs in 607 samples in pigs. The TFs that highly co-expressed (|Rho| ≥ 0.3 and *P*-value < 0.05) with eRNAs were considered as the potential regulators of eRNAs^23^.

### eRNA-trait analysis

We download the pig quantitative trait loci (QTL) from AnimalQTLdb (https://www.animalgenome.org/cgi-bin/QTLdb/SS/index), and the eRNAs close to 2 Mb regions of QTLs were denoted as QTL-associated eRNAs. QTL enrichment was tested with a Fisher Exact test using an in-house R script, and the *P*-value ≤ 0.05 with the relative enrichment >1 were considered as significant.

### Housekeeping and tissue-specific eRNAs

Considering the temporal activity of enhancers, we further annotated the tissue-specific eRNAs and housekeeping eRNAs. The constitutively expressed eRNAs (RPM ≥ 1) which expressed in all tissues were defined as HKeRNAs, according to previous studies^38,42^. The HKeRNAs were further classified into three groups using the coefficient of variation (CV). Specifically, the CVs of HKeRNAs ≤ first quartile were lowly variable expression, the CVs of HKeRNAs < third quartile and å first quartile were medium variable expression, and the CVs of HKeRNAs ≥ third quartile were highly variable expression. To demonstrate the reliability of our eRNAs, we used the results in the PigGTEx (http://piggtex.farmgtex.org/) to verify the target gene of HKeRNAs and tissue-specific eRNAs. The eRNAs of one certain tissue expressed more than 3 times of the average expression in other tissues were defined as tissue-specific eRNAs^37,38^.

## Data Records

The dataset is available at Figshare:

File 1: This file contains the coordinate of enhancers. The column headings are chromosome number, start site, end site and enhancer ID of enhancer regions. This file can be found in (10.6084/m9.figshare.22923353)^43^.

File 2: The file contains the eRNA regions of 15 tissues in pigs. The column headings are the eRNA id, chromosome number, start site, end site and enhancer id. This information can be found in (10.6084/m9.figshare.22923353)^43^.

File 3: The file contains the sequence similarity analysis between pig eRNAs and human eRNAs. The column headings are pig species, pig eRNA id, reference species, reference eRNA id, identify, evalue, the chromosome number, middle. This information can be found in (10.6084/m9.figshare.22923353)^43^.

File 4: A zip-file compressed tar archive contains the correlation between eRNAs and their target genes in pigs. The column headings are eRNA id, gene id, gene name, Rho and FDR. This information can be found in (10.6084/m9.figshare.22923353)^43^.

File 5: A zip-file compressed archive contains the correlation between eRNAs and regulators in pigs. The column headings are eRNA id, TF id, TF name, Rho and FDR. This information can be found in (10.6084/m9.figshare.22923353)^43^.

File 6: This file contains the tissue-specific eRNAs of 15 tissues in pigs. The column headings are tissue-specific eRNA id, chromosome number, start site and end site. The file can be found in (10.6084/m9.figshare.22923353)^43^.

File 7: A zip-file compressed archive contains the correlation between the tissue-specific eRNAs and their target genes in 15 tissues of pigs. The column headings are eRNA id, gene id, gene name, Rho and FDR. This information can be found in (10.6084/m9.figshare.22923353)^43^.

File 8: This file contains the list of Housekeeping eRNAs with CV. This file can be found in (10.6084/m9.figshare.22923353)^43^.

File 9. The file contains the correlation association analysis of eRNA and QTL. The column headings are enriched trait, eRNA id, *P*-value and estimate. This file can be found in (10.6084/m9.figshare.22923353)^43^.

## Technical Validation

### CNNEE implements two functions: enhancer prediction and eRNA identification in pigs

#### The enhancer prediction module

**Fig. 2:**
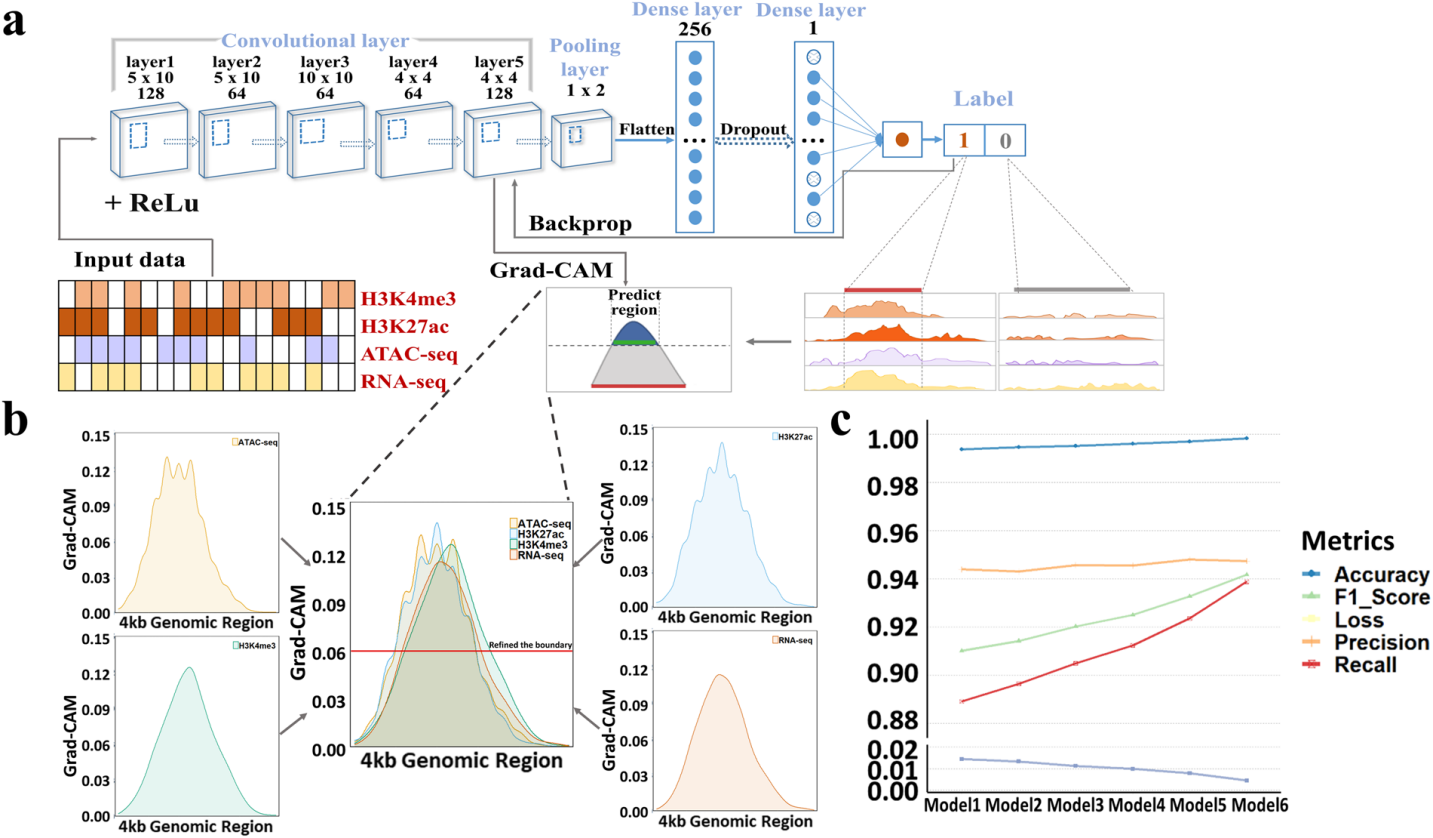
CNNEE Model schematics and metrics. (**a**) The schematics of CNN strategy in CNNEE. (**b**) The schematics of Grad-CAM to refine the boundary. (**c**) The metrics (accuracy, precision, f1 source, recall, loss) of the six models.

To accurately identify enhancers in pigs, the powerful CNN strategy was utilized to construct the binary classifier to characterize the enhancer over a 4 kb sliding window. ATAC-seq peaks overlapping with a transcript peak and the peaks of an active histone enhancer (H3K27ac and H3K4me3) were defined as the active enhancers and positive training regions. The residuals were considered as negative training regions (see Methods). A total of six models with different convolutional and pooling layers were built to train the positive and negative regions (see Methods) (Fig. 2a). To further refine the boundaries of the candidate regions by adjusting the weights, the Grad-CAM method was added to CNNEE (Fig. 2b). To validate the results, we performed five cross-validations by dividing the data into five folders and reusing each folder as an out-of-sample validation set. We found Model 6 achieved the highest optimal metrics with Accuracy: 0.9983, Precision: 0.9474, Recall: 0.9388, F1 source: 0.9417 (Fig. 2c) and selected for further predict enhancers in pigs. The accuracy of CNNEE was higher than that of REPTILE^14^ (0.9550) and DECODE^16^ (0.9897). Moreover, if we deleted the transcriptomic data from CNNEE, the F1 score decreased to 0.9032, and the recall rate decreased to 0.8786. This observation demonstrated that the transcriptomic data were powerful in improving the predicted accuracy of enhancers. To further investigate the accuracy and resolution of CNNEE pipeline, we applied it on human and mouse liver database from ENCODE^44^ (Supplemental Table S2), and found CNNEE achieved the Accuracy: 0.9976 and Precision: 0.9437 for mice, coupling with Accuracy: 0.9966 and Precision: 0.9435 for humans. Notably, 70.7% (humans) and 71.3% (mice) of the predicted enhancers by CNNEE (Figshare File 1) overlapped and conserved with enhancers reported in humans and mice of ENCODE, respectively. These results indicated that our CNNEE pipeline showed a wide availability and practicality in mammals, suggesting the predicted enhancers were reasonable and plausible in pigs.

#### The eRNA identification module

To depict the atlas of eRNAs in pigs, we collected 607 RNA-seq samples from 15 tissues including hearts, livers, spleens, backfat, gallbladder, jejunums, kidneys, lungs, ovaries, pituitaries, muscles, Sk-Muscles, Ld-Muscles, pineal and ileum. We defined the eRNAs with average RPM ≥ 1 in at least one tissue as the detectable eRNAs (Figshare File 2). In addition, about 81.4% of pig eRNAs were sequence-conserved with human eRNAs in HeRA^23^ (Figshare File 3).

### Validation of the enhancers and eRNAs functional characteristics

**Fig. 3:**
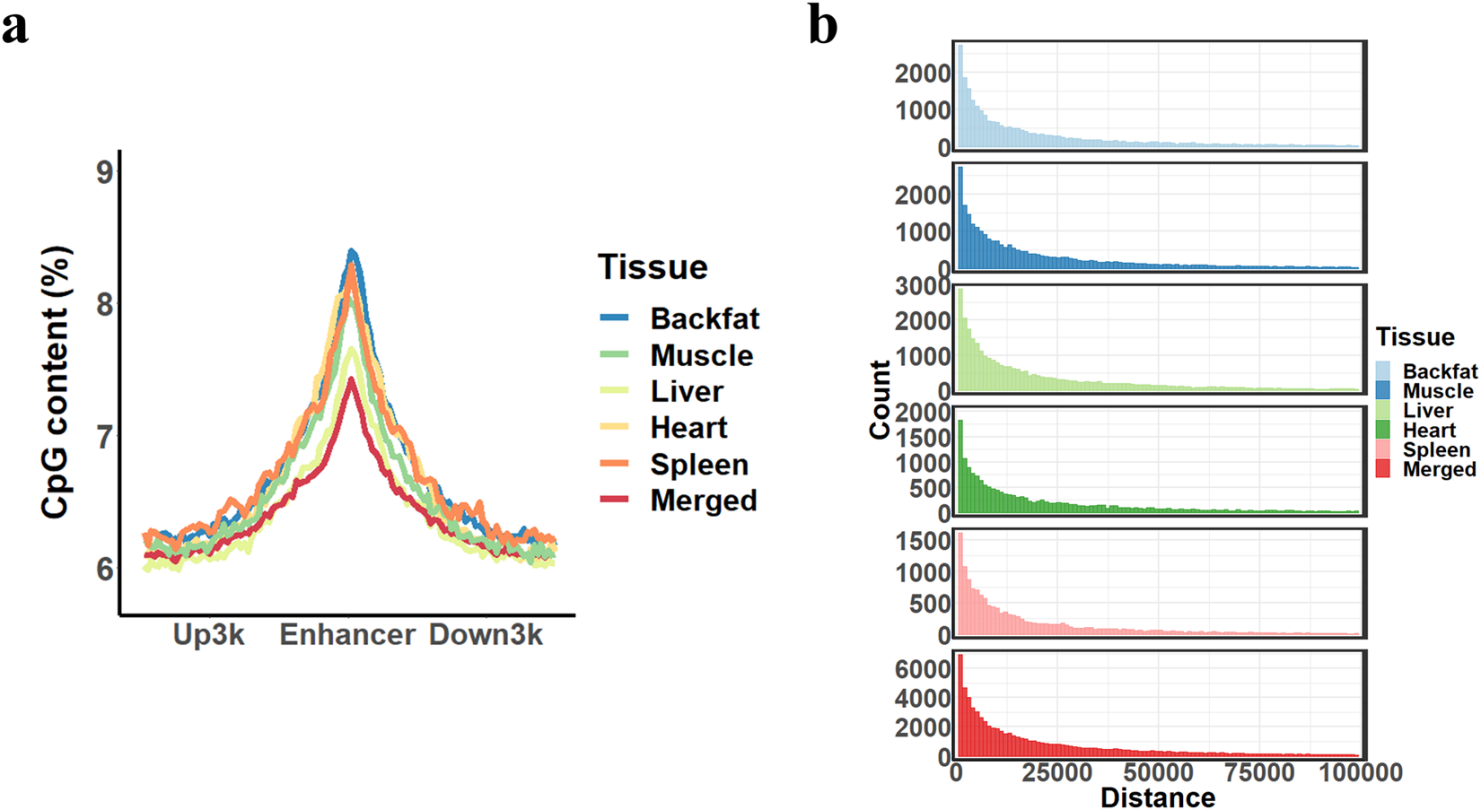
Enhancers across five tissues of pigs. (**a**) The CpG density patterns of enhancers; (**b**) The distances of enhancers to the nearest TSSs.

The CpG density of the central region of enhancers was obviously higher than flanking regions (Fig. 3a), and 84.7% of the enhancers were less than 50 kb away from the nearest TSS (Fig. 3b). The average length of candidate enhancers was refined to 539 bp in length, which was 31% shorter than Zhao *et al*.^39^ and 19% shorter than Pan *et al*.^45^. To further validate enhancers, we downloaded the peak files of H3K4me1 of porcine livers from Pan *et al*.^45^, which were not used in the training model of CNNEE, and found that 83.5% of enhancers predicted by CNNEE were overlapped with the marks of H3K4me1. To verify the conservation of the pig enhancers, we compared pig and human enhancers, and the results showed that 83.8% of the enhancer regions were sequence-conserved with human genome (hg38) (LiftOver, minimum match = 0.5), and 82.0% of sequence-conserved enhancers were functionally conserved with human enhancers of ENCODE, indicating that the characterized enhancers were pinpointed in pigs.

To analyze the differences between conserved enhancers and non-conserved enhancers in terms of sequence and function. We found the CpG densities of conserved enhancers were significant higher than that of non-conserved enhancers (*P*-value = 0.0020) (Supplemental Figure S1). The average number of target genes (Figshare File 4) regulated by conserved enhancers was significant less than that of non-conserved enhancers (*P*-value < 2.2e-16), and the average number of TFs (Figshare File 5) that regulated the expressions of eRNAs for conserved enhancers was significant higher than that of non-conserved enhancers (*P*-value = 0.026). Moreover, we found that the tissue-specific eRNAs were significantly enriched in non-conserved enhancers (*P*-value < 2.2e-16, relative enrichment = 1.17).

**Fig. 4:**
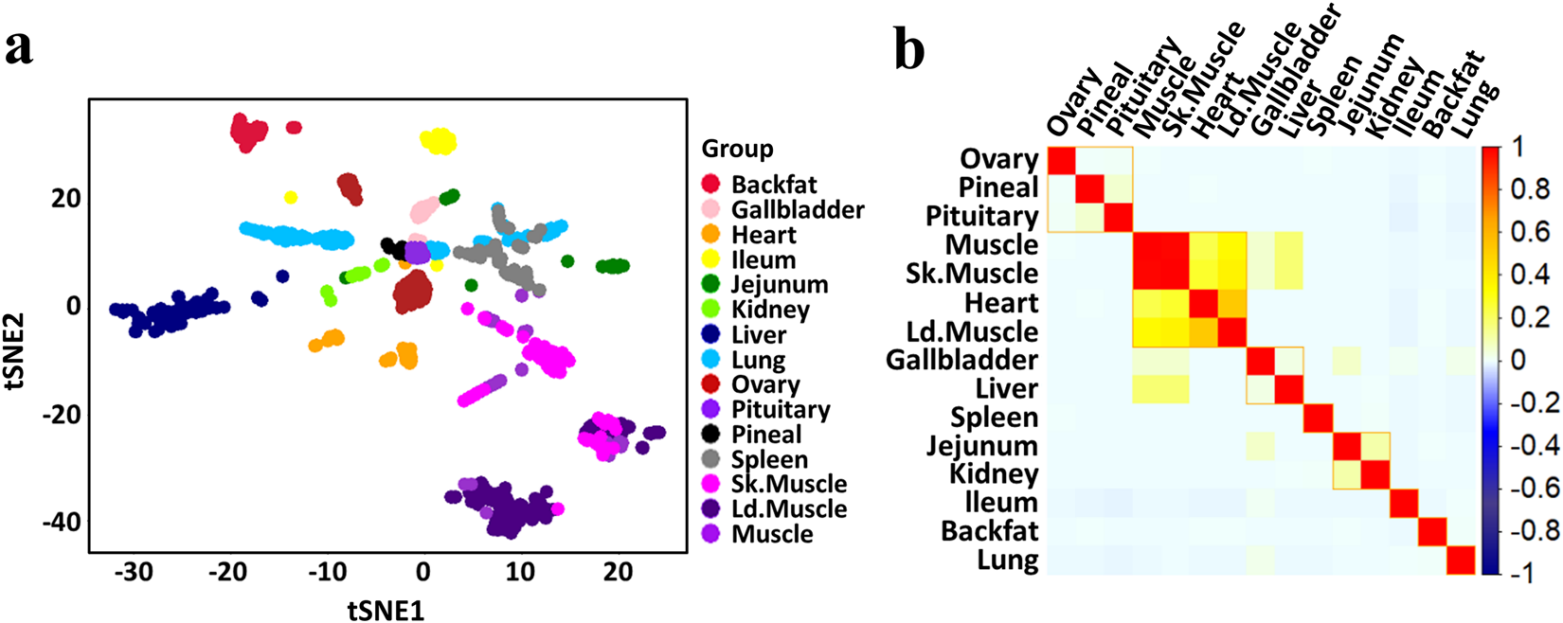
The tissue-specific eRNAs across 15 tissues of pigs. The tSNE of tissue-specific eRNAs using the per sample expression in each tissue (**a**). The heatmap of tissue-specific eRNAs using the average expression in each tissue (**b**).

To validation the biological functions of eRNAs across pig tissues, we determined the tissue-specific eRNAs in 15 tissues (Figshare File 6) and performed GO and KEGG enrichment analysis of the target genes (Figshare File 7) for tissue-specific eRNAs. Tissue-specific eRNAs with similar physiological function were more likely to cluster together, as revealed by tSNE and heatmap cluster analysis of tissue-specific eRNAs (tSNE and heatmap, Fig. 4a,b, respectively). Supplemental Figure S2 displayed several significant GO terms that are enriched (*P*-value <  = 0.05) in tissue-specific eRNAs correspond to its known tissue-related biological functions. For example, the biological functions of ovary-specific eRNAs were enriched in female gonad development (GO: 0008585), development of primary female sexual characteristics (GO: 0046545), female sex differentiation (GO: 0046660), ovulation cycle process (GO: 0022602), development of primary male sexual characteristics (GO: 0046546). Notably, we found that the conserved enhancers were likely to maintain the fundamental biological functions, and the non-conserved enhancers appeared to keep the tissue-specific functions of livers (Supplemental Figure S3). Moreover, we classified HKeRNAs into three groups with low, medium, and high expression variability using thresholds of first quartile and third quartile of CV (Figshare File 8). Supplemental Figure S4 summarized the GO and KEGG enrichment analysis of HKeRNAs with low and medium variable expression, and the results show that the target genes are mainly involved in the basic biological activities of the organism, e.g., chromatin silencing (GO:0006342), regulation of gene expression, epigenetic (GO:0040029) and notch signaling pathway (ssc04330). We used pigGTEX (http://piggtex.farmgtex.org) to query the expression of target genes, 63.2% of the target genes for HKeRNAs were stably expressed in pigGTEX. For example, the *SNRPC*, *IDH2*, *EMC1* and *POP7* were the target genes of HKeRNAs, and Supplemental Figure S5 showed their expression in 34 tissues of pigGTEX.

To validate the relationship between tissue-specific eRNAs and phenotypes, we downloaded publicly available pig QTLs from AnimalQTLdb. (https://www.animalgenome.org/cgi-bin/QTLdb/SS/index). The eRNAs close to 2 Mb regions of QTLs were denoted as QTL-associated eRNAs for 670 traits. We found that the eRNAs were significantly enriched in the QTL regions (*P*-value < 2.2e-16, relative enrichment = 3.36), indicating the regulatory roles of eRNAs were powerful. 652 traits were associated with these tissue-specific eRNAs (Figshare File 9).

### Supplementary information

Supplementary Figure

Supplemental Table S1

Supplemental Table S2

Supplemental Table S3

## Data Availability

All CNNEE code for enhancer prediction and eRNA identification is publicly available at https://github.com/WangYF33/CNNEE.
