# Supplementary material for: Pig-eRNAdb: a comprehensive enhancer and eRNA dataset of pigs
Source: Sci Data. 2024 Feb 1;11:157. doi: 10.1038/s41597-024-02960-7 (PMC10834423; doi:10.1038/s41597-024-02960-7)
Supplement: Supplementary file 1 — Supplementary Figure [file 41597_2024_2960_MOESM1_ESM.pdf]

Supplemental Figures

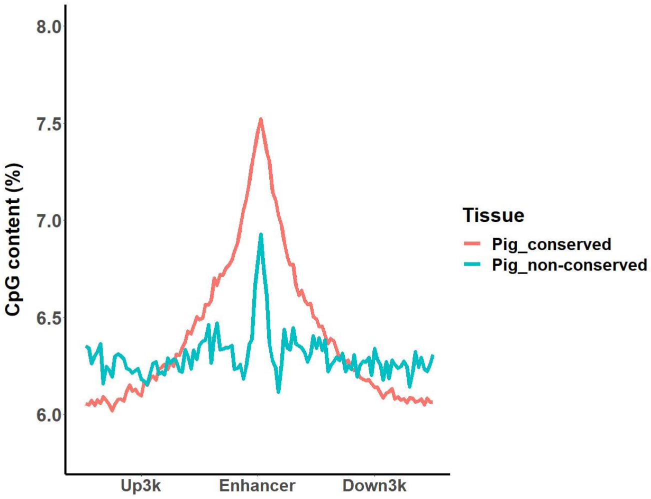

Figure S1: The CpG density patterns of conserved and non-conserved enhancers.

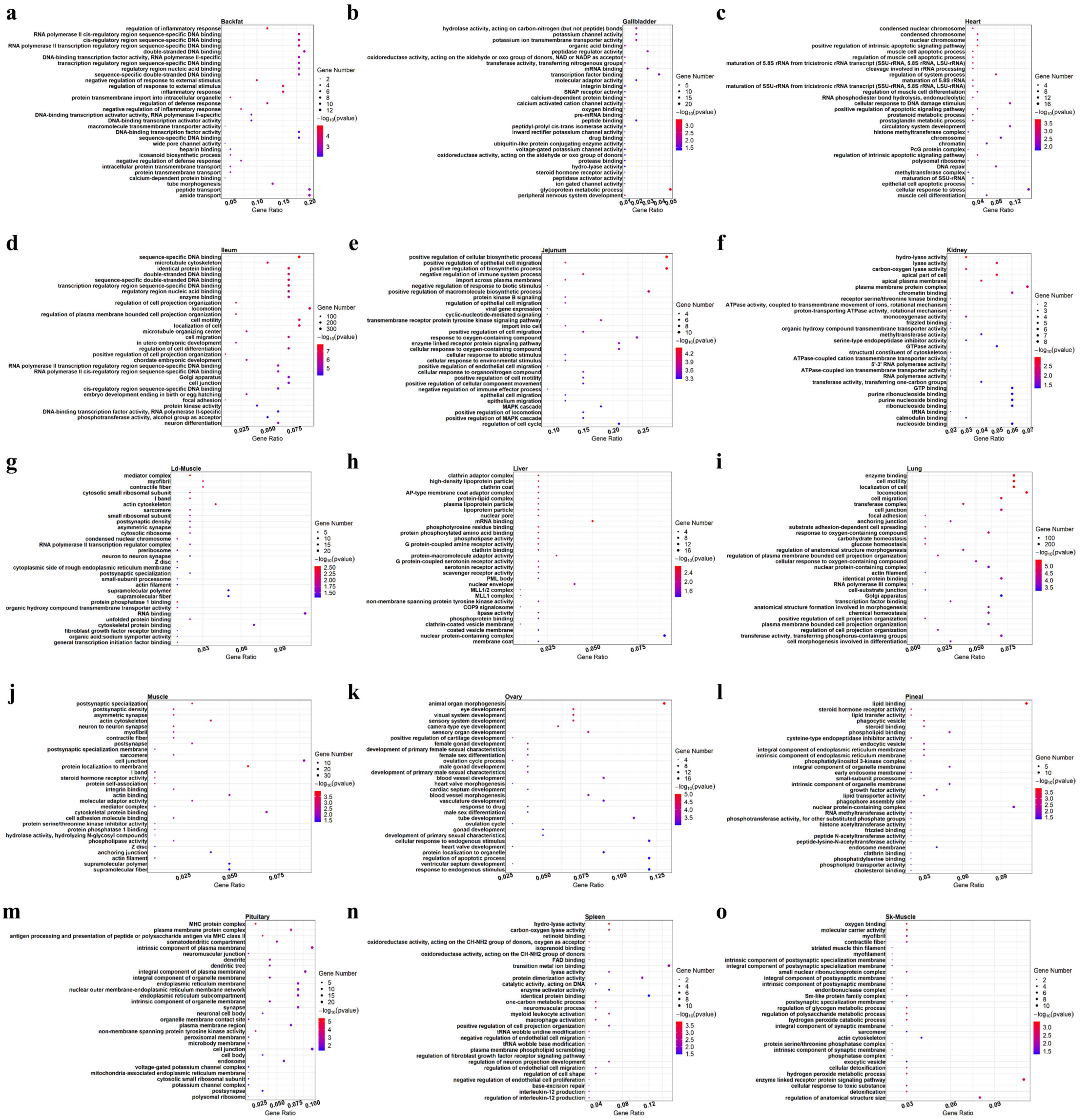

Figure S2: The GO enrichment analysis of the target genes for tissue-specific eRNAs in 15 tissues, e.g. backfat (a), gallbladder (b), heart (c), ileum (d), jejunum (e), kidney (f), Ld-muscle (g), liver (h), lung (i), muscle (j), ovary (k), pineal (l), pituitary (m), spleen (n), Sk-muscle (o).

a

b

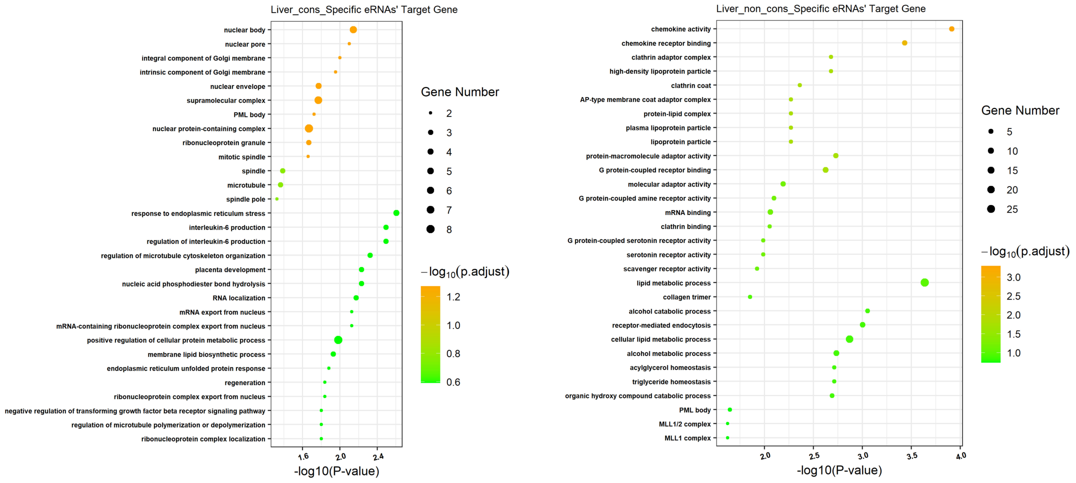

Figure S3: The GO enrichment analysis of the target genes for conserved (a) and non-conserved (b) enhancers in liver.

a

b

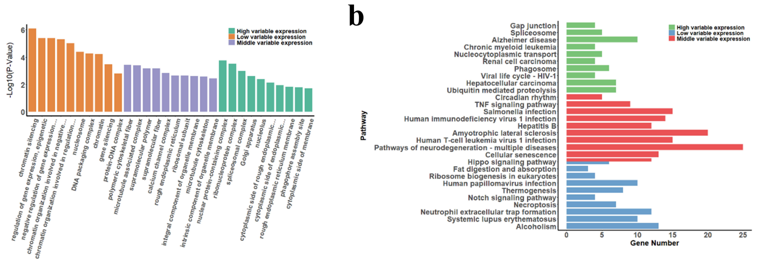

Figure S4: The HKeRNAs and tissue-specific eRNAs across 15 tissues of pigs. The GO (a) and KEGG (b) analysis of low variable expression, medium variable expression, and high variable expression of HKeRNAs.

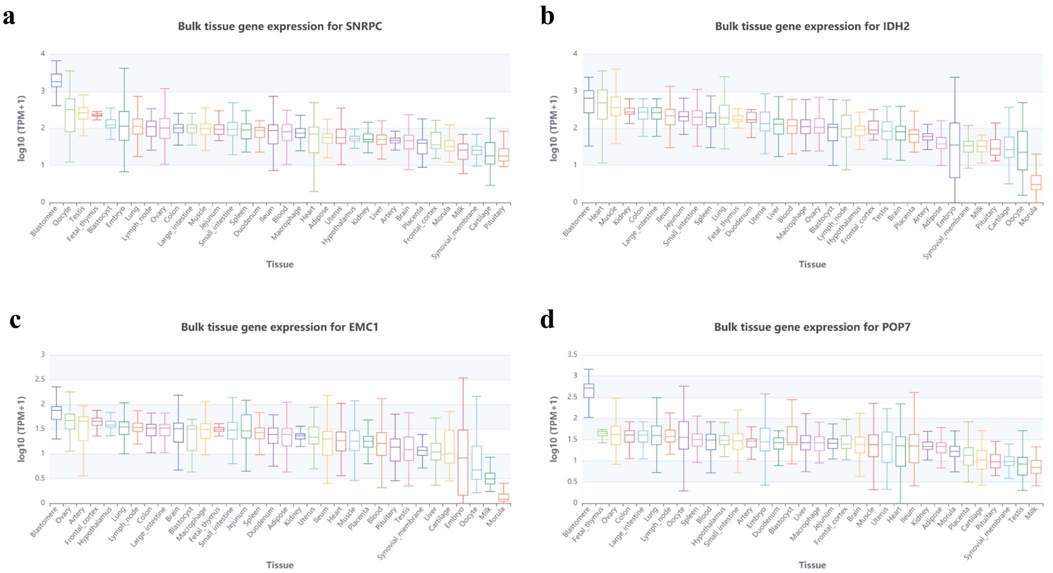

Figure S5: The expressions of four target genes for HKeRNAs in PigGTEx. (a) The expression of SNRPC gene in PigGTEx. (b) The expression of IDH2 gene in PigGTEx. (c) The expression of EMC1 gene in PigGTEx. (d) The expression of POP7 gene in PigGTEx.
